# Complete genome sequences of nine *Rhodococcus equi* phages

**DOI:** 10.1128/mra.01088-23

**Published:** 2024-01-05

**Authors:** Myles D. Radersma, Gabrielle Lathrop, Karena C. Moleakunnel, Luke A. Harlow, Aerin E. Baker, Alison J. Chen, Jason G. Churu, Carly A. Dole, Sophia L. Doorn, Ethan M. Hill, Anna Howland, Amanda Janvier, Catie M. Kramer, Matt J. Minasian, Jocelyn R. Nieze, Isabella K. Perezrios, Fiona J. Ramsey, Katie L. Seinen, Sierra K. Swierenga, Michael M. Veenstra, Grace E. Weaver, Alexandra C. White, Esther Yoon, John T. Wertz, Randall J. DeJong

**Affiliations:** 1 Department of Biology, Calvin University, Grand Rapids, Michigan, USA; Department of Biology, Queens College, , New York, USA

**Keywords:** bacteriophages, *Rhodococcus*, genomes, Actinobacteria

## Abstract

We report genomes of nine phages isolated from Actinobacteria *Rhodococcus equi* NRRL B-16538. Six of these phages belong to actinobacteriophage cluster CR, which otherwise contains *Gordonia* phages; two form the CF cluster; and one is a singleton. Genome lengths are 62,017–80,980 bp with 63.9%–67.3% GC content.

## ANNOUNCEMENT

*Rhodococcus equi* is an Actinobacteria found in soil and known to cause lung infections in animals, including foals and cattle, and immunocompromised humans (1) and for which few phages have been characterized (2, 3). Here, we report on nine phages isolated in 2022 using *R. equi* NRRL B-16538, using standard methods (4). Soil samples collected from ~5 cm below the soil surface were washed in peptone-yeast extract-calcium chloride (PYCa) broth before being filtered (0.22-µm pore size) (Table 1). Directly plating the filtrate in PYCa top agar with *R. equi* yielded phages Apiary and Polyyuki. Incubation of the filtrate with *R. equi* for 2 days at 30°C before refiltration and plating yielded Jflix2, Shagrat, Braxoaddie, GuyFagieri, Maselop, MacGully, and Reynauld. All phages have siphovirus morphologies, as determined by transmission electron microscopy (Table 1).

**TABLE 1:** . Characteristics of nine phages isolated from *Rhodococcus equi* NRRL B-16538*.*

Phage name	Soil sample collection site	Plaque morphology	Plaque size (mm)^*a*^	Capsid size (nm)^*b*^	Tail length (nm)^*b*^	Average shotgun coverage (fold)	Genome length (bp)	Genome ends	G + C content (%)	No. of ORFs	Cluster	GenBank accession no.
Jflix2	Grand Rapids, MI, 42.927083 N, 85.588702 W	Round and hazy	1–2	80–90	300	1,635	67,017	CCCGCT 3′ single-stranded overhang	66.5	106	CF	OQ938594
Shagrat	Grand Rapids, MI, 42.933306 N, 85.581389 W	Round and hazy	0.5–1.0	60	270	3,415	62,204	CCACT 3′ single-stranded overhang	63.9	114	CF	OQ938576
Apiary	Grand Rapids, MI, 42.933934 N, 85.591096 W	Round and clear	1	55–73	255	4,019	67,424	AGCCGCGTAC 3′ single-stranded overhang	67.4	96	CR	OR434026
Braxoaddie	Grand Rapids, MI, 42.932919 N, 85.597268 W	Round and clear	1–2	70–80	308	1,933	67,415	AGCCGCGTAC 3′ single-stranded overhang	67.3	95	CR	OQ938585
GuyFagieri	Grand Rapids, MI, 42.932919 N, 85.58709 W	Round and clear	1	60–70	290	2,480	67,084	AGCCGCGTAC 3′ single-stranded overhang	67.1	93	CR	OR434027
MacGully	Caledonia, MI, 42.817778 N, 85.546667 W	Round and clear	1–2	70–80	220	3,670	66,998	AGCCGCGTAC 3′ single-stranded overhang	66.2	102	CR	OR283216
Maselop	Grand Rapids, MI, 42.930070 N, 85.589005 W	Round and clear	1	70	285	1,719	67,419	AGCCGCGTAC 3′ single-stranded overhang	67.3	95	CR	OR475250
Polyyuki	Grand Rapids, MI, 42.933366 N, 85.58658 W	Round and clear	1	60	275	3,660	67,447	AGCCGCGTAC 3′ single-stranded overhang	67.3	94	CR	OR475276
Reynauld	Grandville, MI, 42.893611 N, 85.738611 W	Round and clear	2	86	375	3,099	80,980	362-bp direct terminal repeat	66.5	103	Singleton	OR159659

^*a*^ Minimum of three plaques measured.

^*b*^ Minimum of two phage particles measured.

Phage DNA was extracted from a lysate following zinc-chloride precipitation and using a Qiagen DNeasy kit, then prepared for sequencing using the NEBNext UltraII-FS DNA library prep kit. Sequencing was performed with an Illumina MiSeq (v.3 reagents), yielding 150-base single-end reads that were assembled using Newbler (v.2.9) and Consed (v.29) to generate contigs with at least 1,635-fold shotgun coverage (5–7). Genomes were then assigned to clusters based on gene content similarity (GCS) (8) to phages in the actinobacteriophage database (https://phagesdb.org/). Genomes were then annotated using DNA Master (v.5.23.6) (9); PECAAN (v.20221109) (10); Glimmer (v.3.02) (11); Genemark (v.2.5) (12); Phamerator (13); BLAST searches against the PhagesDB and National Center for Biotechnology Information (NCBI) nonredundant databases (14); and HHPred searches against the PDB mmCIF70, Pfam-A, UniProt-SwissProt-viral, and NCBI Conserved Domain Database (15). Scanning genomes with Aragorn (16) and tRNAscanSE (v.2.0) (17) revealed no tRNA-coding regions. Membrane domains in putative proteins were identified with DeepTMHMM (v.1.0.24) (18) and TOPCONS (v.2.0) (19). All software were run using default parameters. Sequencing details and genomic characteristics are provided in Table 1.

Seven out of nine phages are most similar to phages isolated on strains of *Gordonia*. Apiary, Braxoaddie, GuyFagieri, MacGully, Maselop, and Polyyuki are assigned to cluster CR, which otherwise contains phages isolated on *Gordonia* strains. Reynauld is classified as a singleton based on limited GCS to any phage in the actinobacteriophage database but shares nearly half of its genes with phages in clusters CS, CX, or DF, which were all isolated on *Gordonia* strains. The two other phages, Jflix2 and Shagrat, form a new cluster, CF, with phage REQI (GenBank: JN116825), also isolated on *R. equi*. Cluster CF phages share little GCS with phages outside this cluster (maximum 3.82%). Jflix2 and Shagrat are predicted to be temperate based on the presence of a putative serine integrase and immunity repressor in each genome. Host range of all nine phages appears to be narrow, resulting in no plaques when plating lysates with titers of 7 × 10^9^ to 1 × 10^12^ PFU/mL on *Rhodococcus globerulus* American Type Culture Collection (ATCC) 15903, *Rhodococcus jialingiae* ATCC 31636, *Gordonia rubripertinctus* ATCC 25593, or *Gordonia terrae* 3612.

## Data Availability

Annotated genome sequences can be accessed for Jflix2, Shagrat, Apiary, Braxoaddie, GuyFagieri, MacGully, Maselop, Polyyuki, and Reynauld at GenBank accession numbers OQ938594, OQ938576, OR434026, OQ938585, OR434027, OR283216, OR475250, OR475276, and OR159659, respectively. Sequence reads are deposited at National Center for Biotechnology Information under Sequence Read Archive accession numbers SRX21368065, SRX21368084, SRX21368069, SRX21368091, SRX21368060, SRX21368068, SRX21368073, SRX21368079, and SRX21368083.
